# Shaping Streamflow Using a Real-Time Stormwater Control Network

**DOI:** 10.3390/s18072259

**Published:** 2018-07-13

**Authors:** Abhiram Mullapudi, Matthew Bartos, Brandon Wong, Branko Kerkez

**Affiliations:** Department of Civil & Environmental Engineering, University of Michigan; Ann Arbor, MI 48109, USA; abhiramm@umich.edu (A.M.); mdbartos@umich.edu (M.B.); bpwong@umich.edu (B.W.)

**Keywords:** smart cities, smart water systems, wireless sensor networks, stormwater, real time control

## Abstract

“Smart” water systems are transforming the field of stormwater management by enabling real-time monitoring and control of previously static infrastructure. While the localized benefits of active control are well-established, the potential for system-scale control of watersheds is poorly understood. This study shows how a real-world smart stormwater system can be leveraged to shape streamflow within an urban watershed. Specifically, we coordinate releases from two internet-controlled stormwater basins to achieve desired control objectives downstream—such as maintaining the flow at a set-point, and generating interleaved waves. In the first part of the study, we describe the construction of the control network using a low-cost, open-source hardware stack and a cloud-based controller scheduling application. Next, we characterize the system’s control capabilities by determining the travel times, decay times, and magnitudes of various waves released from the upstream retention basins. With this characterization in hand, we use the system to generate two desired responses at a critical downstream junction. First, we generate a set-point hydrograph, in which flow is maintained at an approximately constant rate. Next, we generate a series of overlapping and interleaved waves using timed releases from both retention basins. We discuss how these control strategies can be used to stabilize flows, thereby mitigating streambed erosion and reducing contaminant loads into downstream waterbodies.

## 1. Introduction

Burdened by aging infrastructure, growing populations and changing hydrologic conditions, many municipalities struggle to adequately manage stormwater [1]. Flash flooding can occur when stormwater infrastructure is unable to convey runoff away from developed areas [2]. At the same time, pollutants from urban runoff—such as nutrients, heavy metals and microbes—can contaminate downstream waterbodies, damaging aquatic habitats and resulting in toxic algal blooms [1]. Traditionally, civil engineers have addressed these challenges by building larger storage and conveyance infrastructure (e.g., basins and pipes). However, this approach suffers from a number of important disadvantages. First, new construction is expensive, and is often unfeasible for chronically underfunded stormwater departments [3]. Second, static designs are inflexible to future changes in weather, population growth, and regulatory requirements [2]. Third, overdesigned conveyance systems can cause flooding, erosion and damage to downstream property and ecosystems, which ultimately necessitates further remediation and construction [1]. In the face of increasing urbanization and more frequent extreme weather events [4,5], new strategies are needed to ensure effective management of stormwater.

In contrast to traditional *steel-and-concrete* solutions, real-time control has emerged as a novel means to improve the performance of stormwater systems at minimal expense. Drawing on wireless communications, low-power microcontrollers, and modern advances in control theory, these systems achieve performance benefits by reconfiguring water infrastructure in real time [1,6]. Real-time control of stormwater basins, for instance, can improve water quality following a storm event by enhancing removal of contaminants [1]. Similarly, active regulation of discharges through constructed wetlands can improve water quality and rehabilitate aquatic habitats [6,7]. More broadly, by controlling flows over a large network, operators can harness the latent treatment capacity of many distributed stormwater assets, effectively turning urban watersheds into distributed wastewater treatment plants [1,6].

A small number of studies have evaluated the benefits of real-time stormwater control. Most of these studies describe retrofits of isolated sites for rainwater capture and on-site pollutant treatment. Middleton and Barrett (2008) show that equipping existing retention basins with real-time controllers can reduce stormwater pollutant loads downstream by increasing the retention time of captured stormwater [8]. Roman et al. (2017) describe an adaptively-controlled rainwater harvesting system in New York City that captures 35–60% more rainwater than conventional systems [9]. Similarly, Klenzendorf et al. (2015) describe a rainwater harvesting pilot project and a retention basin retrofitted for real-time control in Austin, Texas [10]. The authors show that the controlled retention basin reduces deposition of nitrogen and total suspended solids (TSS) into the downstream system. These studies demonstrate that active control can significantly improve the performance of existing sites at a lower cost than new construction. However, benefits are only examined at a local scale. This distinction is important, given that localized practices do not necessarily achieve the best system-scale outcomes. Indeed, some research indicates that when local best management practices are implemented without accounting for global outcomes, they can produce adverse flow conditions at the watershed scale [11].

Currently, the benefits of coordinated stormwater control are poorly understood. Inspiration for the benefits of system-level control can be taken from sewer operations. While most sewer systems still only rely on local control logic, such as water level setpoints [12], recent work has demonstrated how wider benefits can be achieved through the cooperative action of multiple controllers working in tandem. The cities of Copenhagen and Barcelona, for instance, implement a combination of local rule-based control, and some higher-level optimization that jointly coordinates actions between groups of actuators [13]. Montestruque and Lemmon (2015) describe CSOnet, a sewer control network consisting of 120 sensors and 12 actuators in the city of South Bend, Indiana [3]. This network uses dynamic control algorithms to adaptively balance hydraulic loads throughout the sewer’s interceptor lines, ultimately reducing combined sewer overflows (CSOs) by as much as 25%. While these systems achieve impressive system-scale control of a large sewer networks, it is still unclear how lessons learned from these proprietary sewer control approaches may translate to the broader control of urban watersheds and separated stormwater systems.

In this study, we describe an approach for managing stormwater discharges across an urban watershed using internet-connected valves and sensors. We show that by actively coordinating releases from two parallel retention basins, we can produce desirable flow regimes at a target location downstream, which would not be possible with passive infrastructure alone. This study takes place in four phases. In the first phase, we describe the development of a real-time stormwater control system in the city of Ann Arbor, Michigan. Building on an existing wireless sensing and control network described in Bartos et al. (2018) [6], we demonstrate how static retention basins can be retrofitted with internet-controlled valves, and present a new method for controlling these basins using a controller scheduling application. In the second phase, we characterize the ability of the control network to shape the downstream hydrograph by releasing impulses of different sizes from two retention basins and determining the magnitude, travel time, and decay envelope of the resulting waves. In the third phase, we use the data gathered from this exploratory analysis to determine the control input needed to produce a flat hydrograph at the outlet of the watershed. We discuss how this control strategy can be used to prevent erosion and reduce phosphorus loads into downstream waterbodies. Finally, in the fourth phase, we show how control inputs can be timed to produce synchronized and de-synchronized pulses at a downstream target location. In addition to demonstrating the precision of the control system, this experiment shows how interleaving pulses can be used to free up capacity in upstream retention basins without inducing synchronized flashy flows downstream. We discuss how these simple control “building blocks” can be used by system operators to achieve more sophisticated stormwater management targets. Unlike most existing systems, our control network uses an open-source hardware and software stack, making it freely available to municipalities that are interested in implementing their own smart stormwater control systems. Thus, when combined with supplementary *how-to* documentation on open-storm.org, this study provides the foundation for an “operator’s manual” for real-time control of urban watersheds.

## 2. Study Area and Technologies

### 2.1. Study Area

This study focuses on a wireless control network in the Mallets creek watershed—an urbanized creekshed located in the city of Ann Arbor, Michigan. This creekshed has been the focus of ongoing efforts to reduce peak flows and improve water quality [14]. The creekshed has an area of about 26.7 km${}^{2}$ and contains streams that altogether exceed 16 km in length. These streams drain into the Huron River and ultimately the Great Lakes. With high areas of development and over 33% imperviousness, little natural land is available for infiltration and uptake, resulting in flashy flows that erode stream banks and result in unstable habitats. These rapid flows drive stream erosion and increased transport of sediments and nutrients out of the watershed [14]. While there are no lakes in the creekshed, there are several natural and manmade stormwater basins that have been constructed to help stabilize flows throughout the creekshed and mitigate the impacts of non-point source runoff.

**Figure 1:**
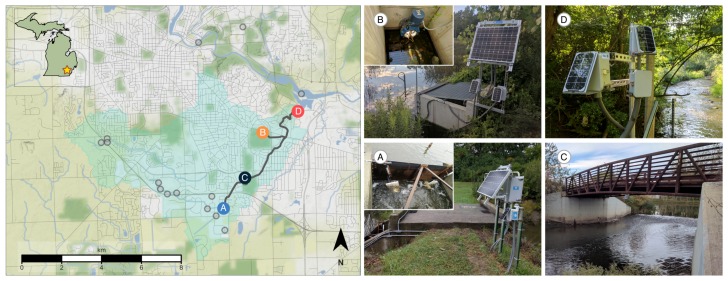
Overview of the study area. The map (**left**) shows the location of relevant control and sensor sites, additional sensor sites (light grey), flow paths between each site (dark grey), and the contributing area of the watershed (light blue). Site images (**right**) show the two control sites (**A** & **B**) along with two downstream sensor locations (**C** & **D**).

To investigate the effects of real-time control on the creekshed, we deploy a control network that measures and regulates flows from two large stormwater basins. The control network consists of four sites centered around the main stem of the creek. Figure 1 shows the locations of each of these four sites in the control network. Water first flows into a large retention basin with a storage capacity of 19 ML (site A), located at the most upstream point in the control network. From this retention basin, water travels 1.4 km downstream to a constructed wetland (site C), designed to slow the flow of water and remove contaminants. After passing through the wetland, water travels another 3 km until it is joined by flows arriving from a smaller retention basin with a storage capacity of 7.5 ML (site B). The combined flows exit the creek at the outlet of Mallet’s creek (site D), after which they enter the Huron River. Internet-controlled valves are deployed at the two stormwater basins at sites A and B. These valves are used in subsequent experiments to regulate flows at the outlet of the creek.

### 2.2. Technologies and Architecture

Flows throughout the creekshed are measured and controlled using a custom wireless sensing and control network. This network is built using the open storm hardware and software stack, which has been described and documented in Bartos et al. (2018) [6]. The hardware layer uses an ultra-low power ARM Cortex-M3 microcontroller (Cypress PSoC), which implements the sensing and control logic in its firmware. Internet connectivity is achieved using a CDMA cellular modem (Telit DE910), which facilitates wireless bi-directional communication between the field device and a remote server. The full unit is powered using a solar-rechargeable 3.7 V lithium-ion battery. To measure the hydrologic response of the system, wireless sensor nodes are deployed along the main stem of the creek. Each sensor node is equipped with an ultrasonic depth sensor (Maxbotix MB7384) to measure water levels (shown in Figure 1, site C). At the time of writing, sensor nodes can be constructed using less than $500 USD of parts.

To control discharges throughout the creekshed, stormwater basins are retrofitted with one of two valves: (i) a 0.3 m diameter butterfly valve (Dynaquip MA44) (Figure 1, site B) or (ii) a 0.3 m gate valve (Valterra 6912) mated to a linear actuator (AEI 6112CH) (Figure 1, site A). Each control valve is connected to a sensor node. The valves are actuated by the microcontroller and powered by rechargeable 12 V sealed lead-acid batteries. Solar panels allow the control sites to operate without line power. Assuming that the valve can be attached to a basin’s outlet without structural modification, each control site can be constructed using less than $3500 USD of parts at the time of writing.

**Figure 2:**
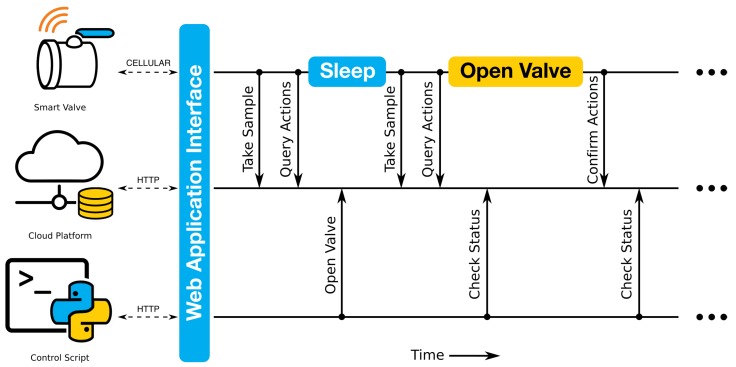
Control system architecture. Field-deployed nodes use a polling system to download and execute commands issued from a remote server. Control actions can be specified manually, or through automated web applications and scripts.

Remote control of valves and sensors is implemented using a *polling* scheme, in which field-deployed nodes request commands from a remote server (Figure 2). To conserve power, nodes spend most of their time in a deep sleep state, consuming only 1–10 μA of current. Upon waking up, each node takes sensor readings and transmits the readings to a cloud-hosted time series database (InfluxDB) via authenticated (and optionally encrypted) *HTTP* requests. Before going back to sleep, the node polls a set of commands from a dedicated feed in the same database. The commands may include, but are not limited to, changing the sampling frequency, triggering additional sensor readings, or opening a valve. Operations can be cancelled and rescheduled either by the application or by an operator. This is useful if, for example, the application detects that a control action was not successfully executed and that pending operations need to be rescheduled. Most importantly, the database supports modern web service standards and application programming interfaces (APIs), which allow the control logic to be quickly implemented via simple web applications. These applications can be written in any number of popular programming languages (Python, Matlab, etc.). This feature improves flexibility, reduces reliance on low-level firmware updates, and allows for the seamless integration of external data sources, such as public weather forecasts [6,15].

For the experiments described in this study, field devices in the creekshed are controlled using a simple Python web application. This application can be executed in either automatic or manual mode. In automatic mode, the application queries water level sensor feeds, rainfall forecasts, and external flow measurements from a publicly-listed measurement station at the outlet of the creekshed (USGS 04174518 (https://waterdata.usgs.gov/usa/nwis/uv?04174518)). Based on these sensor readings, new commands are then written to the database to open and close valves. In manual operation, a predefined set of commands is written to the database, then subsequently executed by the field device. For this study, the manual operation mode is used. The application toolchain is implemented on an Amazon Web Services (AWS) medium-sized linux Elastic Compute Cloud (EC2) instance.

## 3. Characterizing Control Actions

**Figure 3:**
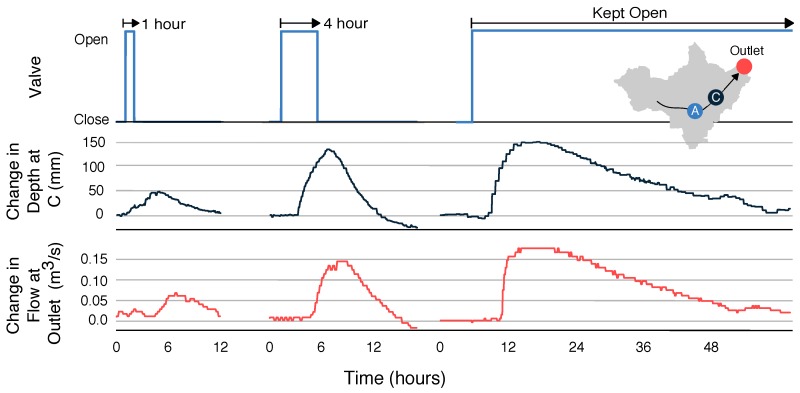
Characterization of control actions from site A. In the first two experiments, the valve at site A is opened for 1-h and 4-h durations. For the third experiment, the valve is held open indefinitely. The resulting waves travel through a constructed wetland (site C) before arriving at the outlet of the watershed. Wave depth (black line) is measured at the wetland, while flow rate (red line) is measured at the outlet.

Before evaluating potential control strategies, we first characterize the ability of each control site to shape downstream flows. Specifically, we quantify the travel time *P* and decay time *D* of various waves as they move between the originating control site and the outlet of the watershed. The characterization is accomplished by releasing pulses of different durations from each stormwater basin and then observing the resulting waves that these pulses generate downstream. To limit confounding effects caused by rainfall, these experiments are carried out during dry conditions (at least four days following a storm). Figure 3 shows a 1-h release, 4-h release, and 48-h release from retention basin A (shown left to right, respectively). The 48-h release empties the retention basin, meaning that this release characterizes the maximum possible output from site A. The travel times for each wave from site A to site C are approximately 3.5 h (time to start of rise) and 6–8 h (time to peak), with faster rise times for the larger releases due to nonlinearities in the speed of wave propagation. The decay times for each release are 6 h, 18 h and 44 h, respectively. From this experiment, it can be seen that the maximum change in flow that site A can generate at the outlet is roughly 0.17 m${}^{3}$/s. Similar experiments are used to characterize site B. From these experiments, we estimate average travel times from site B to the outlet of 1.5 h (time to start of rise) and 1.8 h (time to peak), with an average decay time of 3 h, and a maximum change in flow of approximately 0.2 m${}^{3}$/s.

**Figure 4:**
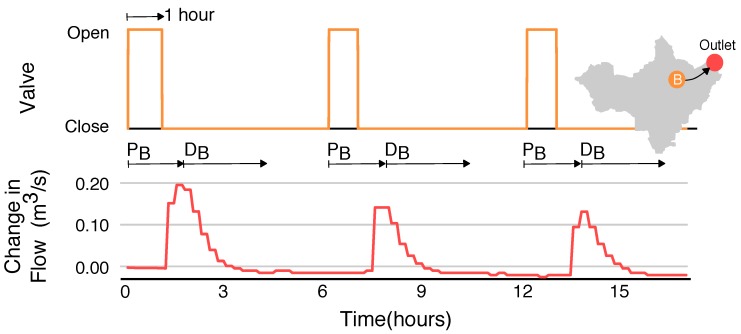
Characterization of control actions originating from site B. Three subsequent pulses are released. While the duration of each control pulse is the same (1 h), the magnitude of the flow at the outlet decreases because the hydraulic head (pressure) in the basin is reduced with each release.

In addition to release duration, sites are also characterized with respect to the hydraulic head (water level) of the originating retention basin. Figure 4 shows the result of releasing three 1-h pulses from site B, without allowing the basin to refill between releases. While the same duration is used for each release, the hydraulic head (stored volume) of the retention basin decreases with each pulse. Thus, the resulting wave becomes smaller with each successive opening of the valve, even though the same input signal is used. In spite of this difference, the travel times and decay times of the waves remain consistent between each release. The magnitude of the resulting wave varies from roughly 0.2 m${}^{3}$/s to 0.13 m${}^{3}$/s, depending on the water level in the basin.

Although retention basin B is significantly smaller than retention basin A, it can produce a comparable change in flow at the watershed outlet (approximately 0.2 m${}^{3}$/s). This effect can be attributed to two main factors. First, site B is located closer to the outlet (3.0 km as opposed to 5.9 km for site A), meaning that the wave is subject to less hydraulic dispersion. Second, the retention basin at site B is elevated higher above the receiving stream, meaning that flows exit the control structure more rapidly than flows released from site A. Thus, compared to site A, site B produces short pulses with a rapid onset and large peak. Despite its relatively smaller volume, control actions from site B must thus be tailored to avoid generating flashy flows at the outlet.

One crucial result of these experiments is that for the purposes of control, nonlinearities in wave propagation can be safely ignored. Shallow-water waves exhibit a nonlinear relationship between wave height and wave speed, meaning that larger waves propagate faster [16]. If these nonlinearities were significant, then control strategies would need to account for changes in travel time due to (i) variations in release durations; (ii) variations in basin head; and (iii) superposition of waves originating from different locations. For the system examined in this study, the effect of these nonlinearities is small. Namely, while nonlinearities in wave propagation affect the shape of the resulting hydrograph (skewing the peak toward the left), they do not significantly affect the bulk travel time of an isolated wave. Specifically, the travel times for site A and site B remain consistent (3.5 h and 1.5 h, respectively) despite scheduling releases of different durations and magnitudes. This result is consistent with findings from previous studies that use linear dynamics for stormwater system control [17,18,19]. Thus, for the scale of our creekshed the travel time of a wave originating at an upstream stormwater basin can be considered independent of both the amount of water released and the water level of the originating basin. Moreover, superposition of two waves from two parallel sources does not effect a noticeable change in bulk wave speed. This result suggests that for the purposes of control, the channel network may be approximated as a linear system in which waves originating from each retention basin can be superimposed in order to produce a desired output hydrograph downstream.

By characterizing the downstream response to various impulsive inputs, these initial experiments yield a set of “building blocks” that are subsequently used to achieve more complex control objectives at the watershed outlet. While the propagation of waves within a channel network is described by nonlinear equations, we find that a linear system approximation adequately describes the dynamics needed to generate control strategies. Thus, the characterization experiments described in this section are conceptually analogous to quantifying the unit impulse response of a linear system. This framework suggests that desired waveforms can be generated via simple linear combinations of known input signals. With this conceptual model in hand, we carry out a number of control experiments to showcase the utility of the stormwater control network. First, we show how pulse-width modulation of a valve can be used to produce a flat hydrograph that meets but does not exceed a given flow threshold. Next, we show how valve releases can be timed to generate synchronized and desynchronized waves at the outlet. These experiments provide recipes for managing releases from upstream retention basins while simultaneously fostering desirable flow conditions downstream.

## 4. Set-Point Hydrographs

Real-time control can be used to flatten downstream hydrographs, helping to reduce erosion and maintain healthy aquatic ecosystems. In passive stormwater systems, hydrographs often exhibit a distinct peak, preceded by a rapid rise and followed by a slower decay. While typically associated with rain events, this phenomenon can also be observed when water is released from a retention basin (see Figure 3 and Figure 4). Peak flows that exceed downstream capacity will often lead to flooding. Furthermore, urban streams can become unstable if a critical flow velocity or flow rate is reached [20]. Exceedance of these thresholds may lead to ecological damage and stream erosion, as well as the mobilization of sediments. These sediments in turn may carry nutrients, metals and other pollutants downstream, impairing water quality and promoting the growth of algal blooms [21]. This particular impairment underpins the major challenge of “urban stream syndrome”, forcing many cities to spend millions of dollars to reduce downstream flow rates [22,23]. While active control has been proposed as a means to condition stormwater flows, the specific control strategies needed to achieve stable flow conditions within an urban watershed are currently not well understood.

To address this challenge, a sequence of control actions is designed to yield a constant set-point condition at the outlet of the watershed. Specifically, we aim to create a flat hydrograph, for which the flow rate remains close to (but does not exceed) a specified value. While the set-point used in this experiment is chosen arbitrarily, this threshold may be chosen to control for objectives related to downstream flooding and water quality—for instance, ensuring that the critical flow threshold for sediment transport is not surpassed. To achieve a constant set-point flow rate, we derive inspiration from *pulse-width modulation*—a method used in electrical systems to generate analog signals from discrete digital pulses. Isolated pulses of water are emitted from the control site, spaced apart such that the arrival time of each wave overlaps with the receding limb of the prior wave. As the pulses travel through the channel network, they disperse, causing the individual waves to overlap and combine. The resulting superposition of partly-dispersed waves results in an approximately constant flow rate.

**Figure 5:**
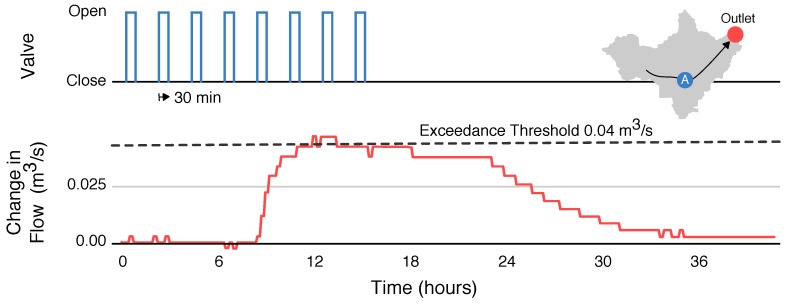
Generating a set-point hydrograph. Small, evenly spaced pulses (30-min duration) are released from the controlled basin. The pulses disperse as they travel through the 6 km-long stream, leading to a relatively flat response at the outlet of the watershed.

As seen in the hydrograph response (Figure 5), the “flat hydrograph” objective is achieved by modulating the valve position in successive 30-min pulses. The flows at the outlet remain approximately flat, without significantly exceeding a setpoint of 0.04 m${}^{3}$/s. Of course, the shape is not perfectly flat, given the large distance between the two sites and nonlinearities inherent in wave propagation. However, these experimental results show that active modulation of a valve can produce highly stable flow conditions downstream that would not be possible using passive infrastructure alone. In a real-world scenario, this control strategy could be used to drain a watershed as fast as possible without exceeding critical flood conditions downstream. Minimizing the change in flows downstream also reduces the likelihood of stream erosion. From our prior studies in this creekshed that were not affected by real-time control [24], it can be estimated that pollutant concentrations during this flat stage were no greater than 127 mg/L for sediment and 0.209 mg/L for total phosphorus. For comparison, keeping the valve open would have resulted in concentrations of at least 390 mg/L for sediment and 0.618 mg/L for total phosphorus. By modulating the valve position to achieve a relatively flat and steady outflow, the control actions likely reduced the total mass of solids and phosphorus that would otherwise contribute to ecological damage and harmful algal blooms. Future studies will confirm and refine these estimates by measuring real-time water quality changes that result from control.

## 5. Coordinated Releases between Multiple Control Sites

**Figure 6:**
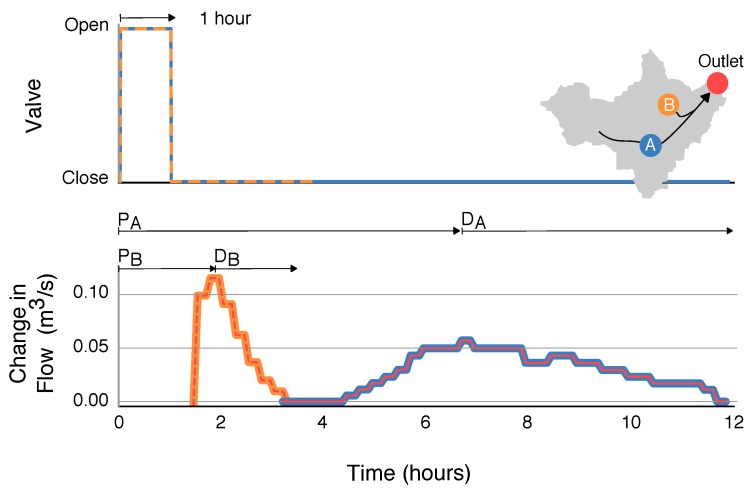
Flows at outlet of watershed resulting from 1 h releases from each control site. Time to peak *P*, magnitude, and decay time *D* for each release are labeled.

Motivated by the larger goal of watershed-scale control, a final experiment is devised to evaluate the level of precision that can be achieved when coordinating releases from multiple sites. Namely, we schedule releases from the two controlled basins in order to produce synchronized and interleaved pulses at the outlet. Before running the experiment, we first determine the control signals needed to generate the combined and interleaved waves, respectively, by assessing the travel time and decay time of waves released from each retention basin. Figure 6 shows the hydrographs resulting from 1-h pulses released simultaneously from site A and site B. Based on the travel times of each wave, it can be seen that in order to achieve a synchronized wave at the outlet, a 1-h release from site B must be scheduled approximately six hours after a 1-h release from site A. Conversely, to achieve an interleaved pattern at the outlet, the following pulse train can be used: (i) release a 1-h pulse from site A; (ii) release a pulse from site B approximately 12 h later; (iii) release a pulse from site A after waiting an additional four hours; and (iv) repeat the pattern starting at step (ii).

**Figure 7:**
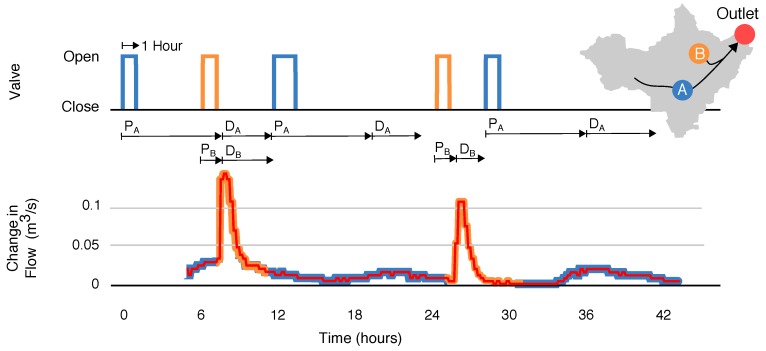
Superposition and interleaving of waves from retention basins A and B. Overlapping waves (coincident peaks) are generated from hours 6 to 12. Interleaved waves (off-phase peaks) are generated from hours 18 to 44.

Once the input signals required to produce each desired shape are known, we schedule a series of commands to be executed by each valve. The experiment is divided into two stages. During the first stage, flows from the control sites are released such that the peaks of the hydrographs overlap. In the second stage of the experiment, the flows are released off-phase, such that the flows arriving from one site begin exactly when the flows from the other site recede. Figure 7 shows the result of this experiment, with the overlapping waves occurring from hours 6 to 15, and the interleaved waves occurring from hours 15 to 44. As hypothesized earlier, the superposition of waves is approximately linear. In other words, the maximum change in flow is approximately equal to the sum of the maximum flow of each component wave. Moreover, the superposition of the two waves does not appear to appreciably change the bulk travel time.

This experiment shows that real-time control of stormwater systems can achieve precise control over downstream flow conditions, and it also suggests a strategy for coordinating releases in order to remove stormwater from retention basins while simultaneously achieving target flow conditions downstream. Like the set-point experiment, an interleaving control pattern can be used to de-water upstream retention basins without exceeding a particular flow threshold downstream. When waves generated by several upstream retention basins combine, they can generate large, flashy flows at a downstream location. This in turn can contribute to erosion of the surrounding channel. For this reason, it is desirable to avoid the collision of waves from two different upstream sources. By interleaving flows from upstream retention basins, one can free up capacity in the system without generating adverse flow conditions downstream. More broadly, the results of this experiment demonstrate the fine level of flow control that can be achieved across urban watersheds using a low-cost sensor and control network. While the underlying control logic only uses rudimentary time-of-travel metrics, it nonetheless produces desirable flow regimes that would be difficult to achieve with passive infrastructure alone. As such, this experiment builds a foundation for more complex control strategies by verifying that the watershed responds consistently and predictably to individual control actions. This result suggests that future studies may one day demonstrate more complex, possibly near-arbitrary, hydrograph shapes. Time of travel may not be sufficient for such approaches, however, and more complex and analytical control techniques should be considered.

## 6. Conclusions

This study shows how internet-connected stormwater control valves can be used to shape streamflows within a large urban watershed. To our knowledge, this study is the first to document how coordinated releases between multiple stormwater control sites can satisfy system-scale watershed performance goals—such as maintaining downstream flow at a constant rate or preventing sediment transport. Building on an existing wireless sensor network, we demonstrate how static stormwater retention basins can be retrofitted with internet-controlled valves to enable active control at a low cost. Characterizing the system in a series of exploratory experiments, we find that a linear approximation is sufficient to describe the downstream response associated with a given input. Next, we use the system to generate two flow conditions downstream: (i) a set-point hydrograph in which flow is maintained at a roughly constant rate; and (ii) a series of overlapping and interleaved waves. We find that pulse-width modulation of upstream valves generates a flat downstream response. Similarly, interleaving of discharges provides an effective tool for emptying upstream retention basins without inducing flashy flows downstream. In addition to demonstrating the precision of the control system, these experiments suggest strategies for managing stormwater transfers across a watershed while maintaining desired flow conditions. To make the smart stormwater system described in this paper accessible to water managers worldwide, all hardware, software and documentation for this project are made available at open-storm.org.

## References

[1] Kerkez B., Gruden C., Lewis M., Montestruque L., Quigley M., Wong B., Bedig A., Kertesz R., Braun T., Cadwalader O. (2016). Smarter Stormwater Systems. Environ. Sci. Technol..

[2] Wright J., Marchese D. (2017). Briefing: Continuous monitoring and adaptive control: The ‘smart’ storm water management solution. Proc. Inst. Civ. Eng. Smart Infrastruct. Constr..

[3] Montestruque L., Lemmon M.D. (2015). Globally Coordinated Distributed Storm Water Management System. Proceedings of the 1st ACM International Workshop on Cyber-Physical Systems for Smart Water Networks (CySWater’15).

[4] Bronstert A., Niehoff D., Bürger G. (2002). Effects of climate and land-use change on storm runoff generation: Present knowledge and modelling capabilities. Hydrol. Process..

[5] Stocker T. (2014). Climate Change 2013: The Physical Science Basis: Working Group I Contribution to the Fifth Assessment Report of the Intergovernmental Panel on Climate Change.

[6] Bartos M., Wong B., Kerkez B. (2018). Open storm: A complete framework for sensing and control of urban watersheds. Environ. Sci. Water Res. Technol..

[7] Mullapudi A., Wong B.P., Kerkez B. (2017). Emerging investigators series: Building a theory for smart stormwater systems. Environ. Sci. Water Res..

[8] Middleton J.R., Barrett M.E. (2008). Water Quality Performance of a Batch-Type Stormwater Detention Basin. Water Environ. Res..

[9] Roman D., Braga A., Shetty N., Culligan P. (2017). Design and Modeling of an Adaptively Controlled Rainwater Harvesting System. Water.

[10] Klenzendorf B., Barrett M., Christman M., Quigley M. (2015). Water Quality and Conservation Benefits Achieved via Real Time Control Retrofit of Stormwater Management Facilities near Austin, Texas.

[11] Emerson C.H., Welty C., Traver R.G. (2005). Watershed-Scale Evaluation of a System of Storm Water Detention Basins. J. Hydrol. Eng..

[12] Schütze M., Campisano A., Colas H., Schilling W., Vanrolleghem P.A. (2004). Real time control of urban wastewater systems—Where do we stand today?. J. Hydrol..

[13] Mollerup A.L., Mikkelsen P.S., Thornberg D., Sin G. (2016). Controlling sewer systems—A critical review based on systems in three EU cities. Urban Water J..

[14] Lawson R., Riggs E., Weiker D., Doubek J. (2016). Total Suspended Solids Reduction and Implementation Plan for Malletts Creek: October 2011–September 2016.

[15] Wong B.P., Kerkez B. (2016). Real-time environmental sensor data: An application to water quality using web services. Environ. Model. Softw..

[16] Kinnmark I. (2012). The Shallow Water Wave Equations: Formulation, Analysis and Application.

[17] Litrico X., Fromion V. (2004). Simplified Modeling of Irrigation Canals for Controller Design. J. Irrig. Drain. Eng..

[18] Marinaki M., Papageorgiou M. Linear-quadratic regulators applied to sewer network flow control. Proceedings of the 2003 European Control Conference (ECC).

[19] Garcia L., Barreiro-Gomez J., Escobar E., Tellez D., Quijano N., Ocampo-Martinez C. (2015). Modeling and real-time control of urban drainage systems: A review. Adv. Water Res..

[20] Bledsoe B.P. (2002). Stream erosion potential and stormwater management strategies. J. Water Res. Plan. Manag..

[21] Michalak A.M., Anderson E.J., Beletsky D., Boland S., Bosch N.S., Bridgeman T.B., Chaffin J.D., Cho K., Confesor R., Daloglu I. (2013). Record-setting algal bloom in Lake Erie caused by agricultural and meteorological trends consistent with expected future conditions. Proc. Natl. Acad. Sci. USA.

[22] Schilling J., Logan J. (2008). Greening the rust belt: A green infrastructure model for right sizing America’s shrinking cities. J. Am. Plan. Assoc..

[23] Wise S. (2008). Green infrastructure rising. Planning.

[24] Wong B.P., Kerkez B. (2016). Adaptive measurements of urban runoff quality. Water Resour. Res..

